# Early rehabilitation with dedicated use of belt-type electrical muscle stimulation for severe COVID-19 patients

**DOI:** 10.1186/s13054-020-03080-5

**Published:** 2020-06-15

**Authors:** Kensuke Nakamura, Hidehiko Nakano, Hiromu Naraba, Masaki Mochizuki, Hideki Hashimoto

**Affiliations:** Department of Emergency and Critical Care Medicine, Hitachi General Hospital, 2-1-1, Jonan-cho, Hitachi, Ibaraki, 317-0077 Japan

**Keywords:** ARDS, Corona virus, COVID, Electrical muscle stimulation, Physiotherapy

Coronavirus disease 2019 (COVID-19), caused by severe acute respiratory syndrome coronavirus 2, has produced a global pandemic. Many patients, mainly those with plural risk factors, have developed to critically ill status and have required intensive care including mechanical ventilation and extracorporeal membrane oxygenation. Although evidence remains limited about how long mechanical ventilation is generally necessary, 1 to 2 weeks of ventilation usage are typically prepared to treat severe cases of COVID-19 [1]. Because intensive care unit (ICU) acquired weakness (ICU-AW) often accompanies acute respiratory distress syndrome, treatment of ICU-AW is fundamentally important for clinical practice for COVID-19.

Early mobilization and exercise are vitally necessary to treat severe COVID-19. In an international expert team’s guide to physiotherapy management for COVID-19, early exercise is recommended, with active involvement by a physiotherapist, after ICU treatment [2]. However, in actual practice, active early rehabilitation can frequently not be performed for various reasons such as exhaustion of medical resources, especially of personal protective equipment. Moreover, Chinese recommendations for respiratory rehabilitation have suggested avoidance of early rehabilitation in the acute phase of severe COVID-19 [3].

To reduce exposure and to conserve medical resources, service automation is also desired in early mobilization. Electrical muscle stimulation (EMS) is an exclusive automated method of physiotherapy used in current critical care. As discussed for EMS efficacy, its use in early acute phase can contribute to the countering of ICU-AW if combined with optimal nutrition therapy including adequate protein delivery [4]. Automated EMS is expected to be an ideal mobilization for severe COVID-19 patients.

Moreover, among the various modes of EMS, belt-type EMS is expected to be effective for critical care because it can induce whole lower extremity exercise through whole muscle contraction between wrapped belts [5]. Exposure to medical staff can be minimized while realizing frequent actuation with a longer duration for each bout of stimulation by assigning each COVID-19 patient a dedicated belt-type EMS, for which no belt change would be necessary (Fig. 1). At Hitachi General Hospital, we assigned each ventilated COVID-19 patients a dedicated belt-type EMS: a measure which achieved better outcomes by administration of 50 min bouts, with three bouts per day, requiring only switching on by a nurse.

**Fig. 1:**
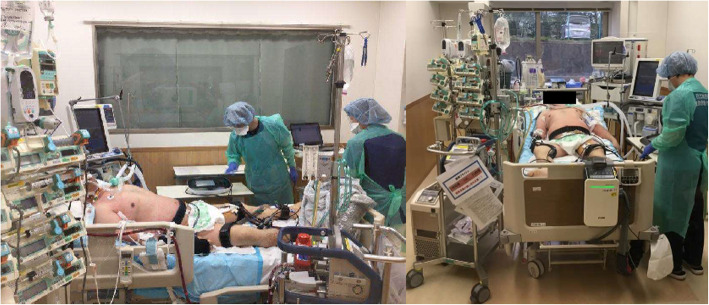
Belt-type electrical muscle stimulation for severe COVID-19 patients. Belt-type electrical muscle stimulation EMS for intensive care unit patient with extracorporeal membrane oxygenation. The EMS machine is dedicated to this patient and is not brought out of the patient’s bedroom. The EMS belt is worn continuously during daytime. This figure was provided after informed consent and permission were received from the patient

In conclusion, we propose the use of dedicated belt-type EMS for early rehabilitation in severe COVID-19. Ultimately, a ventilator with an installed EMS would be ideal, not only for COVID-19 patients, but also for all future ventilated patients.

## Data Availability

Not applicable.
